# Biases in AI: acknowledging and addressing the inevitable ethical issues

**DOI:** 10.3389/fdgth.2025.1614105

**Published:** 2025-08-20

**Authors:** Bjørn Hofmann

**Affiliations:** ^1^ Centre of Medical Ethics, The University of Oslo, Oslo, Norway; ^2^ Institute of the Health Sciences, The Norwegian University of Science and Technology (NTNU), Gjøvik, Norway

**Keywords:** bias, ethics, autonomy, accountability, transparency, transformation, artificial intelligence, machine learning

## Abstract

Biases in artificial intelligence (AI) systems pose a range of ethical issues. The myriads of biases in AI systems are briefly reviewed and divided in three main categories: input bias, system bias, and application bias. These biases pose a series of basic ethical challenges: injustice, bad output/outcome, loss of autonomy, transformation of basic concepts and values, and erosion of accountability. A review of the many ways to identify, measure, and mitigate these biases reveals commendable efforts to avoid or reduce bias; however, it also highlights the persistence of unresolved biases. Residual and undetected biases present epistemic challenges with substantial ethical implications. The article further investigates whether the general principles, checklists, guidelines, frameworks, or regulations of AI ethics could address the identified ethical issues with bias. Unfortunately, the depth and diversity of these challenges often exceed the capabilities of existing approaches. Consequently, the article suggests that we must acknowledge and accept some residual ethical issues related to biases in AI systems. By utilizing insights from ethics and moral psychology, we can better navigate this landscape. To maximize the benefits and minimize the harms of biases in AI, it is imperative to identify and mitigate existing biases and remain transparent about the consequences of those we cannot eliminate. This necessitates close collaboration between scientists and ethicists.

## Graphical Abstract

**Figure:**
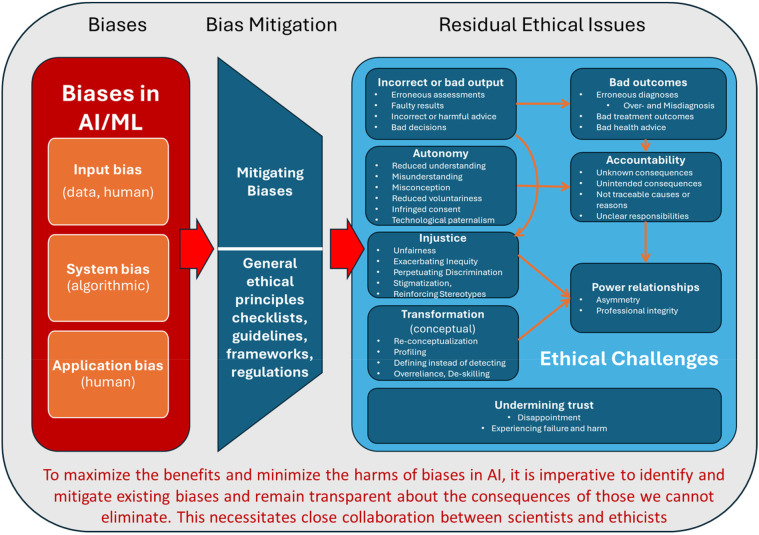

## Introduction

The literature on how to identify and assess biases in artificial intelligence (AI) is burgeoning (1–3). So is the literature on how to mitigate such biases (1, 2, 4–10). However, despite great efforts, the problem prevails. So far, biases cannot be eliminated from AI systems. Some biases we therefore have to live with—including their ethical issues.

Correspondingly, there has been a proliferating literature on the ethics of AI (11–20). A wide range of ethical principles, checklists, guidelines, and frameworks have emerged for addressing basic ethical challenges in AI (12, 14–18, 20–35). However, they are rarely tailored to address the ethical aspects of biases.

Hence, there is a need to scrutinize the ethical aspects of biases in AI in more detail. While some studies have addressed specific ethical issues of bias, such as fairness (36), more comprehensive and elaborate analyses are needed.

Accordingly, this article addresses four key questions:

1. What are the biases identified in AI systems? (short overview)
2. What are the basic ethical issues with biases in AI systems?
3. How can biases in AI systems be identified, measured, and mitigated (in order to avoid or reduce their ethical implications)?
4. What can we do to acknowledge and address these (residual) ethical issues with biases in AI?

Very many biases have been identified in AI systems. However, despite great efforts, not all of them seem amenable to mitigation—some we do not know how to mitigate, and others we might not even recognize. Hence, there appear to be unknown residual biases posing epistemic challenges with ethical implications. This article identifies five inevitable ethical challenges with bias in AI (forming the acronym IBATA): Injustice, Bad output/outcome, Autonomy, Transformation, and Accountability.

That is, bias in AI poses special epistemic challenges which are difficult to eliminate and which has important ethical implications (4, 37–40). Unfortunately, general principles, checklists, and frameworks of AI ethics do not seem to be able to address these ethical issues. Therefore, we must identify and mitigate as many biases as possible and strive to reveal the consequences of those that cannot be avoided. Moreover, we must acknowledge and actively address the inevitable ethical challenges with bias to ascertain that the benefits outweigh the harms. Overall, we must strive to use the powerful tool of AI to obtain our goals instead of letting it dictate our values.

For practical reasons, the scope of this study, and its examples, will be limited to healthcare. While the findings may be relevant for AI bias in general, this warrants a separate study.

Artificial intelligence (AI) is used as a generic term, including machine learning and deep learning.

## Methods

To address the four questions above narrative reviews are conducted to provide overviews of (1) the biases in AI, (2) the ethics principles, guidelines, and frameworks for artificial intelligence (AI), and (3) of the ways to identify, measure, and mitigate biases in AI. Narrative reviews were conducted according to (41, 42). As other such reviews, this narrative review is “non-quantitative, thematic, educational and …. opinionated” (43).

Initial searches for the topics were done in Google Scholar. Supplemental searches were done in PubMed. Logical search terms were “bias* in AI” and “ethic* in AI”. Combinations with “review” and “systematic review” were applied to limit the number of hits. After title and abstract screening, 98 references were included. Snowballing included additional 53 references, and a reviewer suggested additional 19 references (for which I am most thankful).

Data extraction and synthesis: content was extracted from the identified references and synthesized according to the research questions using thematic content analysis. Standard (normative) ethical analysis is applied to identify profound (residual) ethical issues.

## Biases in AI systems (RQ1)

Bias is defined as “pervasive simplifications or distortions in judgment and reasoning that systematically affect human decision making” (44). There is a proliferating literature on biases in AI, and the biases are generally divided in three main types (1, 3, 7, 45–47): *input bias*, *system bias*, and *application bias*.

*Input biases* are biases in the input data for algorithm training. Data can be incomplete, erroneous, or contain biases of a wide range of kinds, e.g., race, sex, age, and socioeconomic status. These biases have many causes, and although they are data-related biases, they originate in human (cognitive and affective) biases, social biases, or organizational biases. Input bias can be revealed by analyzing the data sets. See below. Supplementary Table S1 provides an overview of some major input biases.

*System bias* is bias in the design and development of algorithms. These biases may originate in selection and sampling (data cleaning, imputation, curation, and treatment of outliers) or in processing and validation of algorithms (48, 49). System bias can be identified and measured by process variables. See below. Supplementary Table S2 provides an overview of some major system biases.

*Application bias* (also called deployment bias or human bias) stems from the use of the AI systems in practice and is prone to a wide range of human biases (5, 45). Additionally there is bias drift over time (model drift/decay, concept drift) (8, 50). Application bias can be identified and measured by comparative outcome analyses.

**Figure 1:**
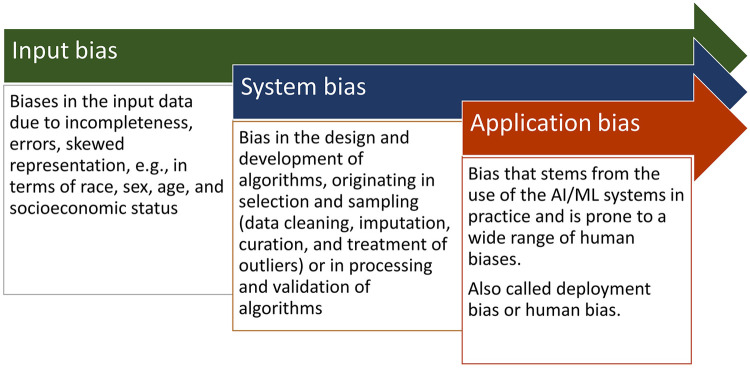
Overview of the three main types of biases in AI.

A recent systematic review showed that the majority of the studies in the healthcare suffered from input bias and system bias (51). Supplementary Table S3 provides an overview of some major application biases. Figure 1 affords an overview of these three types of biases in AI.

Hence, there is an overwhelming number of biases that can appear in AI systems. Let us now turn to the next question: Which ethical issues do they pose.

## Ethical issues with biases in AI (RQ2)

Clearly the vast variety of biases will pose specific ethical issues in particular contexts. However, certain general characteristics of the biases over a variety of contexts may expose some generic ethical issues that are of relevance to a wide range of AI applications. Moreover, while biases may have positive effects, this study will concentrate on its potential negative aspects. The reason for this is that it is crucial that we are aware of and address these in the development, implementation, and use of AI systems.

The most obvious negative implication of bias in AI systems is increased (risk of) *harm* and reduced *safety*, as well as *adverse effects* resulting from erroneous decisions, diagnosis, treatment, or prognosis. The problem is that it violates the ethical principle of non-maleficence (relating to the ancient principle of *primum non nocere*).

Correspondingly, bias may result in poor or erroneous output from the AI system, resulting in bad outcome reducing the *effectiveness* of healthcare services (52). This may origin in a range of the biases listed in S1–3 as well as in model drift, and context ignorance (45). One example is how AI-based tools for assessing skin cancer result in poorer outcomes for populations with diverse skin tones (53). Hence, bias may hamper utility, such as health improvement, and infringe the principle of beneficence.

Yet another obvious ethical challenge following from AI bias is discrimination, unfairness, and stigma. Biases in terms of race, sex, gender, age, socioeconomic status, and ableism are well-documented and undermine the principles of justice and fairness (40, 54–56). As stated in the NIST report “[t]hese biases can negatively impact individuals and society by amplifying and reinforcing discrimination at a speed and scale far beyond the traditional discriminatory practices that can result from implicit human or institutional biases such as racism, sexism, ageism or ableism” (45).

Biases are often latent, that is, they will not be apparent before after long time use (57). This poses a basic *epistemic problem*: the uncertainty of biases adds to the problem of understanding the output from AI systems (explainability, the black-box problem). This challenges the principles of autonomy (and the rule of informed consent) as people are not appropriately informed. It also undermines transparency and accountability.

Several biases can also influence *human agency* as they may reduce human oversight and control, e.g., due to overreliance on AI systems. For example, overreliance on advice has been demonstrated amongst radiologists assessing chest x-rays and making diagnostic decisions (58). Corresponding, the idea that all problems can be solved by technology (techno-solutionism) (59, 60), the belief that technology is always the solution (technochauvinism) (61, 62), or the conception of a technological imperative (63–66), “technological paternalism,” (67, 68) or AI-paternalism (69) may reduce human agency as well as challenging the principle of respect for autonomy.

*Conceptual challenges* raise from biases transforming basic conceptions (70). As pointed out by Floridi: “The digital is deeply transforming reality” (71). Biases may coupling, decoupling, or recoupling features of the world and thereby incite reconceptualization and re-ontologizing of the entities in the world (72). In healthcare this may occur when AI-systems constructed to detect specific conditions (or diagnoses) come to define the same conditions or when AI measures replace human experiences, such as pain or suffering (73). For example, biomarker-based algorithms may change the way we conceptualize, experience, and handle cognitive impairment and Alzheimer's disease. Relatedly, concept drift/creep, model drift, model decay may transform basic conceptions (50). The transformation or re-conceptualization may change social norms and values as well as challenging autonomy and accountability.

Correspondingly, bias may have a *hermeneutic effect*. The output from AI systems may incite new interpretations of agency, personhood, and self-understanding. For example, AI measures may come to (re)define health and disease (wellbeing and suffering) and influence people's interpretation of signs and symptoms, but also of their (self)understanding. This may again challenge their autonomy, integrity, and accountability. It may also instigate hermeneutic epistemic injustice (74).

Moreover, biases may result in a lack of *traceability*, resulting in dissolved or unclear responsibilities (6, 75). Due to lack of transparency in general, and with respect to bias in particular, it can be difficult to hold anybody responsible for errors or harms of bias in AI systems. Again, bias may undermine accountability, and establishing accountability for biased AI outcomes can be difficult (38, 76).

Biases, such as automation complacency (45) or automation bias (5), result in overreliance on AI systems, reduced critical reflection, and deskilling (77, 78). This may change power-relationships and professional integrity, influencing professional ethics. Accordingly, biases in AI systems may reduce trust in such systems and their providers.

Thus, biases in AI systems have a range of ethical implications, raising a series of basic ethical issues, and may undermine several fundamental ethical principles: “the purposes for which AI systems are developed and applied are not in accordance with societal values or fundamental rights such as beneficence, non-maleficence, justice, and explicability” (18). Table 1 provides an overview of ethical implications of AI bias, as well as explanations, examples, and ethical principles or issues arising from these implications.

**Table 1:** Overview of ethical implications of AI bias, explanations, examples, and ethical principles or issues following from these implications.

Implications	Explanation	Example	Ethical principle, issue
Safety	Increased risk, adverse effect/harm	Erroneous decisions, diagnosis, treatment, prognosis	Non-maleficence, negative utility
Effectiveness	Poor output, bad outcome	Does not improve health	Beneficence
Epistemic: Uncertainty	Lack of understanding	Black-box problem Incomprehensibility	Autonomy Transparency, Accountability
Agency	Lack of control, Paternalism	Tech-reliance, Technochauvinism, Quick Fix	Autonomy
Transformative Conceptual challenges	Reconceptualization Defining > detecting	AI measures frame, form, or replace conceptions and experiences, e.g., of Alzheimer's disease	Accountability Social norms and values Integrity
Hermeneutic changes	New interpretations of agency, personhood, (self)understanding	AI measures come to define basic human experiences, such as health and disease	Autonomy, Integrity, Accountability, Epistemic injustice
Justice	Discrimination, unfairness, stigma	Sex, gender, age, race, rurality, education, economy	Justice Fairness
Traceability	Dissolved or unclear responsibility	Nobody can be held responsible for decisions	Accountability Liability
Under-, Overreliance	Automation bias	Inappropriate reliance on AI	Trust Power(lessness)
De-skilling	Reduced critical reflection	Less experience in examination	Professional (competency and) integrity

Hence, a plethora of ethical implications and issues have been identified resulting from biases in AI. Let us now turn to the next question: How can biases be identified, measured, and mitigated? If biases can mitigated, it would resolve or reduce the ethical challenges.

## Identifying and mitigating biases in AI (RQ3)

A wide range of approaches have been developed to identify, measure, and mitigate biases (1–3, 7, 79–81). General checklists, such as STARD-AI, TRIPOD-AI, PROBAST, MI-CLAIM, MINIMAR, TEHAI, DECIDE-AI etc aim at avoiding biases.

Correspondingly, there are many methods for detecting and measuring biases in AI systems, such as equalized odds, statistical parity, Context Association Test (CAT), Word Embedding Association Test (WEAT), counterfactual fairness, predictive parity, Categorial Bias Score (CBS), Embedding Coherence Test (ECT) and others. For example, large chest x-ray data sets can be used to demonstrate underdiagnosis bias of artificial intelligence algorithms in under-served patient populations (82). Table 2 gives a brief overview of general checklists for avoiding biases, measures of bias in AI, and bias measurement data sets.

**Table 2:** General checklists for avoiding biases, measures of bias in AI, and bias measurement data sets.

General checklists for avoiding biases	Measures of bias in AI	Bias measurement data sets
Standards for Reporting of Diagnostic Accuracy Study checklist for AI, STARD-AI	Equalized odds	StereoSet, stereotypical biases in gender, profession, race, and religion
Transparent Reporting of a multivariable prediction model for Individual Prognosis or Diagnosis for AI, TRIPOD-AI	Statistical parity	WinoBias, identify gender bias in coreference resolution systems
Prediction model risk of bias assessment tool (PROBAST)	The Context Association Test (CAT)	BBQ, bias benchmark for question answering
Minimum information about clinical artificial intelligence modeling, CLAIM/MI-CLAIM	Word Embedding Association Test (WEAT)	BOLD: Dataset and metrics for measuring biases in open-ended language generation.
MINimum Information for Medical AI Reporting, MINIMAR	Counterfactual fairness	
Translational Evaluation of Healthcare AI (TEHAI)	Predictive parity	
Stage-specific reporting guideline for the early and live clinical evaluation of decision-support systems based on artificial intelligence, DECIDE-AI	The Categorial Bias (CB) score	
SPIRIT-AI, a set of recommendations for clinical trial protocols evaluating interventions with an AI component (Liu, 2020)	The Embedding Coherence Test (ECT)	
Recommendations for clinical trial reports evaluating interventions with an AI component, CONSORT-AI	Association without ground truth	
Checklist for AI in Medical Imaging (CLAIM)	Natural language inference	
Consolidated Standards of Reporting Trials-Artificial Intelligence (CONSORT-AI)		

Based on (2, 46, 180).

Additionally, there are measures for mitigating biases, such as bias mitigation guidelines (2), checklist (7), bias-handling algorithms (83), debiasing systems, as well as data sets for measuring bias and the effects of bias-mitigating measures (2, 84), as shown in Table 3. Assessing these approaches is beyond the scope of this study, but specific methods can be found in the literature (84). Correspondingly, there are methods to measure and increase fairness by reducing bias (80, 85).

**Table 3:** Guidelines and checklist for mitigating bias, debiasing systems, as well as data sets for measuring bias and the effects of bias-mitigating measures.

Bias mitigation measures or strategies	Bias mitigation impact evaluation data sets
Bias mitigation checklist (7)	Corpus of Linguistic Acceptability (CoLA)
Guidelines: • Creating a well-defined goal • Reviewing the training and input data • Using explainable and interpretable models • Feature selection and pre-processing • Regularized model training • Model validation • Algorithmic auditing, • Hyperparameter tuning • Fairness-aware algorithms • Monitoring and feedback	Stanford Sentiment Treebank (SST-2)
Debiasing devetloped AI systems • Synthetic data augmentation • Re-sampling to balance class distributions • Reweighing to ensure fair representation • Disparate impact remover • Biased embedding correction • Debiasing	Toolkits for detecting and mitigating bias • AI Fairness 360 (AIF360, IBM) • Fairlearn (Microsoft) • What-If Tool (Google) • Fiddler AI

Based on (1, 2, 6, 7, 9, 10, 81).

A recent systematic review of electronic health record-based models revealed that 80% of the identified bias mitigation studies reported improved performance after bias mitigation while 13.3% observed unchanged bias after mitigation, and 6.7% found performance variability based on the applied evaluation metrics (1). More specifically, a reduction of racial bias of 84% has been reported by changing the index variable in a commercial prediction algorithm to identify and help patients with complex health needs (56). Yet another example is how group-based training of algorithms for cardiac segmentation in MRI images substantially reduced bias to a standard deviation of 0.89, while making the algorithm impractical (79, 86). Other studies have shown limitations of explainability tools for bias identification (87).

Typical for many mitigation measures is that they are specific and fragmented. They address explicit issues, such as fairness (36, 40), or are directed towards specific biases or processes of AI development (1). However, they may miss out on a range of specific and overarching biases. Moreover, methods for bias measurement and mitigation may themselves be biased. For example what measure you use to estimate fairness (e.g., Equalized odds, Equal opportunity, Precision-recall parity, Predictive equality, Predictive parity, Equal conditional use accuracy, or Equal selectivity), how you choose to estimate these (in terms of true positive rate, area under receiver operating characteristic curve, false positive rate etc), and whether you correct or normalize the calculations, will influence the assessment of bias and fairness (36).

**Figure 2:**
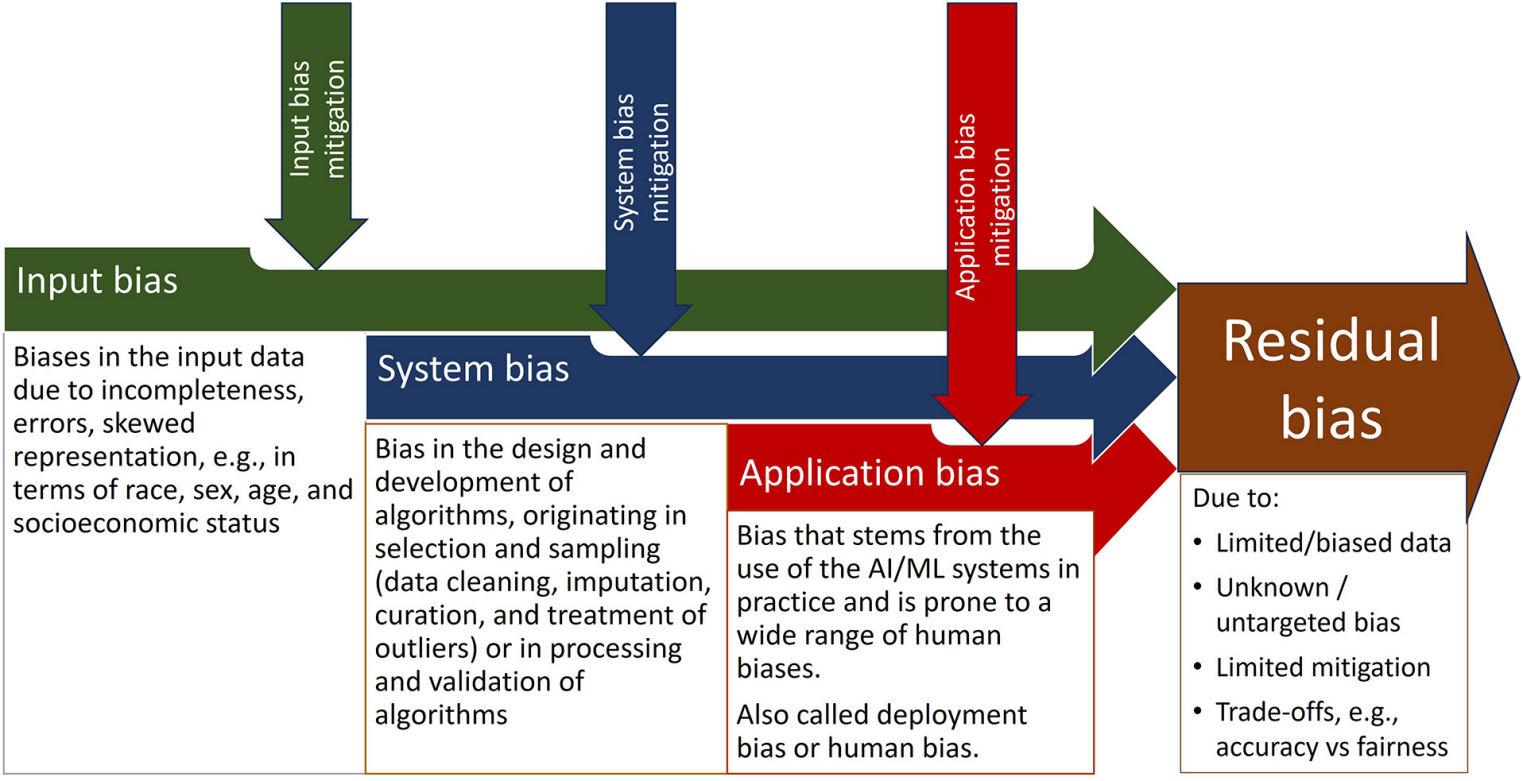
Residual bias after mitigating various types of bias in AI.

Thus, while novel or evolving approaches, such as algorithmic auditing (88) may further reduce bias in AI, so far we have to address the residual biases. See Figure 2.

Moreover, as we do not know what we do not know about biases in AI systems, there are unknown and unavoidable biases. Other biases may be known, but their effect is unknown. They pose Knightian uncertainty (89). Additionally, biases may stem from indeterminacy, as many key concepts such as pain, suffering, and dysfunction can be defined in many ways. Each definition may have its pros and cons—biasing the outcome of AI systems. Thus, despite great efforts to identify and reduce biases in AI systems, they still pose fundamental epistemic challenges with basic ethical implications.

## Acknowledging and addressing ethical issues with biases in AI (RQ4)

How then, can we address the ethical implications of biases in AI? Can they be tackled by applying (some of) the very many ethical principles, approaches, guidelines, checklists, and frameworks that have been developed for ethics in AI? Or do we need other approaches?

### Using general ethical principles to address bias problems

Due to the general ethical concerns with AI a wide range of ethical principles, approaches, guidelines, checklists, and frameworks have been developed (11–18, 34, 90). WHO's ethical principles (20), position papers on AI ethics for trustworthy AI (91), as well as regulations, such as the US Algorithmic Accountability Act (92) and the EU Artificial Intelligence Act (93) are but some examples of such efforts.

**Figure 3:**
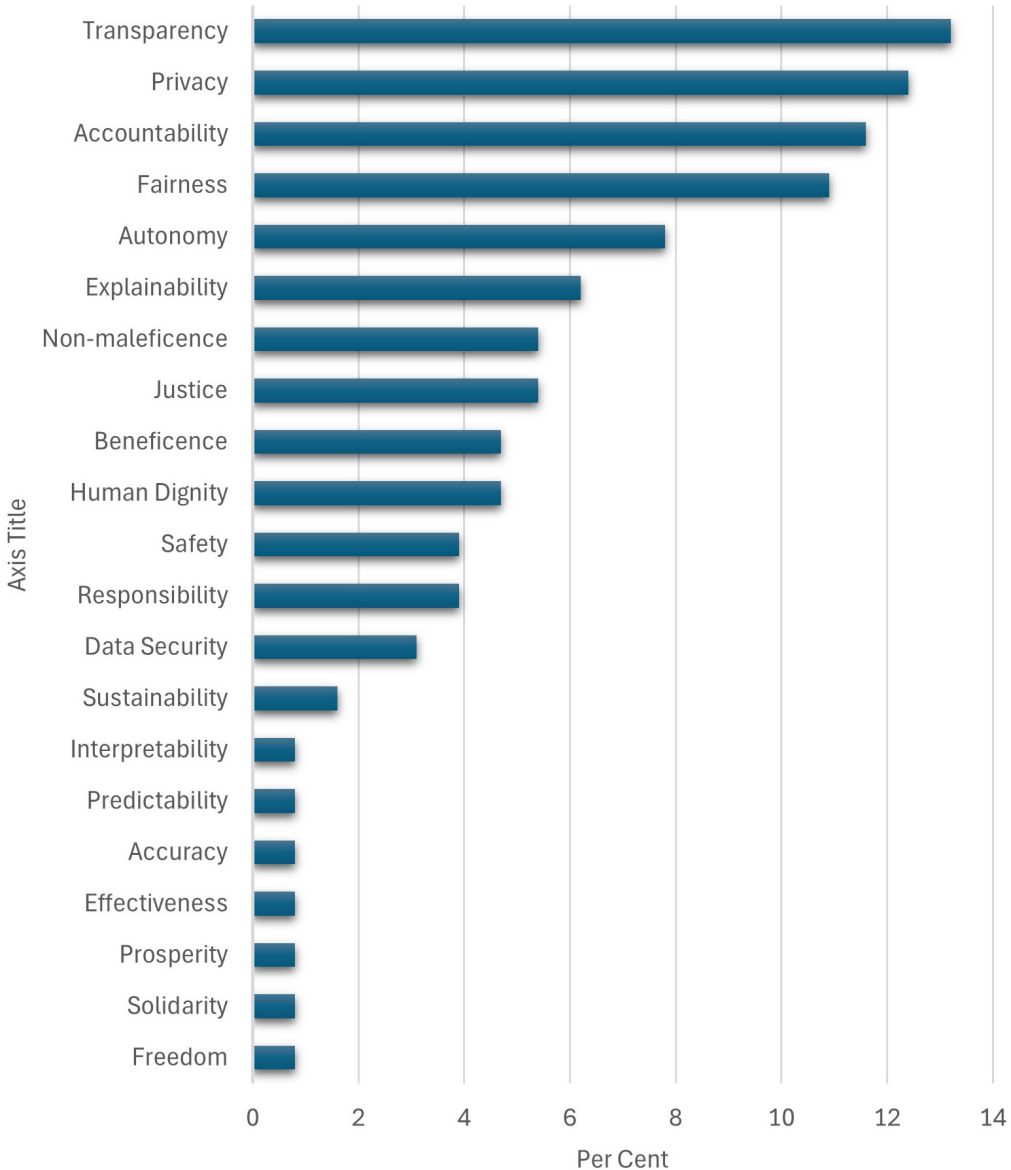
Relative frequency in percent of the AI ethics principles identified in a recent systematic review (14).

Several (systematic) reviews provide good overviews of ethical issues and principles (13, 16, 26, 94–97), as illustrated in Figure 3 (with data from 14).

As can be seen from Table 1, many of the ethical issues and principles at stake for AI in general are relevant with biases in AI as well. For example, transparency and accountability are challenges with bias as well. However, this does not warrant that the general principles or frameworks can address the ethical issues with bias. As pointed out, some biases are latent and unknown while others cannot be eliminated.

Concurrent with the compilation of ethical principles for AI there is an increased awareness of a range of challenges with applying them in practice (14, 98, 99). Vagueness, practical applicability, strong counterforces, and lack of ethical competency are but some of these challenges (14, 100). Additionally, Hagendorff points to other poignant problems in his evaluation of ethics frameworks for ethics in AI. “Currently, AI ethics is failing in many cases. Ethics lacks a reinforcement mechanism. Deviations from the various codes of ethics have no consequences. … Furthermore, empirical experiments show that reading ethics guidelines has no significant influence on the decision-making of software developers. … Distributed responsibility in conjunction with a lack of knowledge about long-term or broader societal technological consequences causes software developers to lack a feeling of accountability or a view of the moral significance of their work. Especially economic incentives are easily overriding commitment to ethical principles and values” (18).

Moreover, Brent Mittelstadt has pointed out that the generally (bioethical) principle-based approach of AI ethics is inadequate as AI development is substantially different from medical ethics as it lacks “(1) common aims and fiduciary duties, (2) professional history and norms, (3) proven methods to translate principles into practice, and (4) robust legal and professional accountability mechanisms” (101). He continues to point out that the real work of AI ethics is “to translate and implement our lofty principles, and in doing so to begin to understand the real ethical challenges of AI” (101).

While it is beyond the scope of this article to investigate all the ethical principles, checklists, guidelines, frameworks or regulations with respect to the very many biases from AI systems, the mentioned shortcomings indicate that such measures cannot solve all the ethical issues following from (unknown or residual) bias. On a positive note, some frameworks are developed to address specific ethical issues (fairness) of bias in AI (36) and for addressing epistemic-ethical issues in the design of AI systems (55), and can be helpful.

Table 4 provides an overview of how various approaches address the five key ethical challenges with biases in AI (forming the acronym IBATA): Injustice, Bad output/outcome, loss of Autonomy, Transformation of basic concepts and values, and loss of Accountability. While several of the frameworks address two or more issues, only one addresses all.

**Table 4:** Overview of whether the established ethical frameworks or principles for AI mention the ethical issues raised by bias. Dark green means that the issue is more or less addressed. White that it is not addressed. Light green that it is mentioned or implicitly addressed.

Challenge framework	Injustice	Bad output/ outcome	Autonomy	Transformation	Accountability
AI4People (102)					
Global landscape (103)					
Machine Ethics (104)					
Dynamics of AI Principles (105)					
IEEE Ethically Aligned Design (EAD) (106)					
The European Commission's High-Level Expert (107)					
Group on Artificial Intelligence Report on the Future of Artificial Intelligence (108)					
OECD Recommendation of the Council on Artificial Intelligence (109)					
The Asilomar AI Principles (110)					
AI Now 2019 Report (111)					
Principles for Accountable Algorithms and a Social Impact Statement for Algorithms					
Montréal Declaration for Responsible Development of Artificial Intelligence (112)					
OpenAI Charter (113)					
ITI AI Policy Principles (114)					
Microsoft AI principles (2025) (114)					
Google and DeepMind Ethics Principles (115)					
Google Perspectives on issues in AI governance (116)					
Everyday Ethics for Artificial Intelligence (117)					
Partnership on AI (118)					

It is also important to notice that very many articles mention ethical principles or frameworks for addressing such issues in AI in general, without demonstrating their application or fruitfulness in the case of bias (83, 119, 120). Others point to ethical challenges with bias (especially fairness) without demonstrating how they can be solved or addressed (121, 122).

### Living with residual bias, epistemic challenges, and prevailing ethical issues

As revealed, biases are abundant in AI systems and raise a range of ethical issues. While some of the biases may be mitigated, residual biases appear to prevail. As such, epistemic challenges will occur. The information, suggestions, and advice from AI systems will sometimes be incorrect or imprecise. The knowledge and the derived evidence will occasionally be uncertain and leave us ignorant about crucial factors. Correspondingly, the measures and concepts applied in AI systems may be vague, ambiguous, and change/drift over time. Hence, the output from AI systems (e.g., diagnoses, treatment suggestions, prognoses, decisions etc) may be wrong. Accordingly, the outcomes from such systems may have uncertain efficacy, effectiveness, safety (123), and efficiency (i.e., cost-effectiveness).

**Figure 4:**
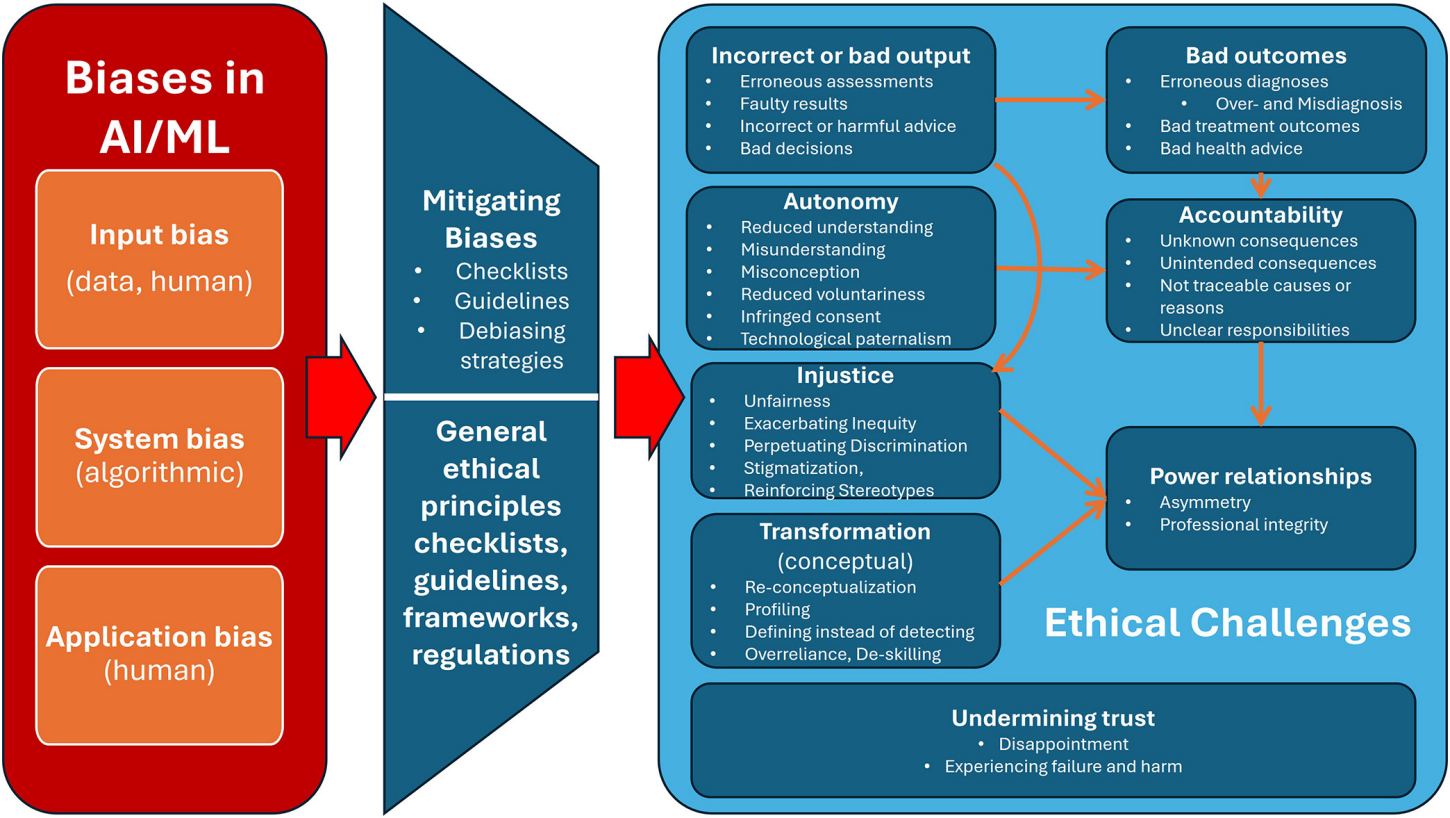
Overview of the main types of biases, mitigating approaches, and basic ethical challenges from residual biases.

This raises a range of ethical issues as elaborated in Table 1. Moreover, they may have regulatory or legal issues (e.g., litigation) and societal challenges (norm creep). Even when applying the rich armamentarium of ethical principles, checklists, guidelines, frameworks, and regulations, some basic issues will prevail: Injustice, Bad output/outcome, loss of Autonomy, Transformation of basic concepts and values, and loss of Accountability (forming the acronym IBATA). Figure 4 sums up the three main types of biases, mitigating approaches, and basic ethical challenges from residual biases.

How then, can we handle the ethical issues following from biases that cannot be mitigated (because they are unknown or because our mitigation measures are insufficient) or addressed by general approaches in AI ethics? Such ethical issues pose genuine moral dilemmas (124), moral distress (125), moral residue (125, 126), moral doubt (127), and even moral injury (128–130). These challenges are not unique to AI and bias in AI, and a range of approaches have been suggested to address moral residue and moral doubt, such as reflective debriefing, professional counseling, and ethics training (131, 132).

Moreover, as the ethical issues from biases in AI stem from epistemic problems (uncertainty and ignorance), measures to handle uncertainty may be relevant. For example, one can apply a range of strategies to develop uncertainty tolerance (133–142), for uncertainty management (143–150), uncertainty handling (151–153), as well as for increasing comfort with uncertainty (154). In particular, strategies to tolerate and manage uncertainty may help with the cognitive, emotional, behavioural, and moral burden of uncertainty of bias in AI systems (both in terms of whether there is bias and its extension). Importantly, it is crucial to avoid *bias numbness*, i.e., that “bias is inevitable, so we need not to care”.

Correspondingly, one can elaborate on basic concepts, such as outcome measures, in order to reduce bias due to indeterminacy and concept creep. For example, ascertaining that outcome measures directly can be related to human pain, dysfunction or suffering (155) (or wellbeing) can avoid biases due to unclear, vague, or biased concepts.

Corresponding to the de-biasing strategies in AI R&D there are many de-biasing strategies for human biases that may be helpful (156–161). Additionally, we need to pay special attention to biases generated by AI, such as overreliance (162), deskilling (77, 78), and acceptance of algorithmic discrimination (162) as they can proliferate or enhance existing bias. Moreover, addressing differences in blaming humans and machines (163, 164) is crucial to address the challenges with accountability.

To maximize autonomy in (biased) AI-based systems it is crucial to be transparent about uncertainty and ignorance about bias and the implications thereof. This is crucial for disclosure in informed consent. Moreover, it is important to be aware of potential paternalism due to bias, e.g., in decision support systems. While paternalism in general is motivated by beneficence, good outcomes may be absent for individuals and groups in biased systems.

To reduce unwarranted transformation, it is crucial to be creative with in envisioning transformative effects of AI systems and their biases. How will the algorithm change our conceptions of the phenomena they handle and the social norms and values that regulate our behavior. For example, biased AI systems for detecting Alzheimer's disease may change our conceptions of cognitive impairment and our social norms and values (and fears) (165). More generally, we should look for potential conceptual changes (related to health and disease, personal identity, and social status) as well as looping-effects, i.e., human adaptation to classifications and altered classifications (166).

Thus, despite ineliminable (residual) bias in AI systems and unavoidable basic ethical issues, there are measures to face with the ethical aspects of bias in AI. The point in this review has been to identify the ethical issues with bias in AI systems (in healthcare) and not to provide a full-fledged framework to address them. This will be the next step. Nonetheless, the review has provided us with some fruitful initial practical guidance for addressing the basic ethical issues of bias in AI systems, summarized in Table 5.

**Table 5:** Summary of the practical implications and guidance for the basic ethical issues of bias in AI systems.

Injustice	Bad output/outcome	Autonomy	Transformation	Accountability
Mitigate bias and its implications as much as possible Be transparent about potential injustice due to bias Make trade-offs between fairness and efficiency transparent	Monitor and audit outcomes from AI-based systems Be transparent about potential differences in outcomes Relate outcomes to basic values, such as reduced pain, dysfunction, and suffering	Be transparent about uncertainty and ignorance about bias and the implications thereof (disclosure) Be aware of potential paternalism due to bias, e.g., in decision support	Be creative with respect to envisioning transformative effects of AI systems and their biases Look for conceptual changes and looping-effects	Clarify responsibility for outcomes Address over-reliance and de-skilling Avoid bias-numbing

Instead of believing that the ethical issues can be avoided or handled by the application of ethical principles or perspectives we have to learn how to face with and live with them. Biases add a new type of uncertainty with ethical burdens, that we have to learn to live with (134). Importantly, the biases make it challenging to ascertain that the benefits from AI applied in healthcare outweigh the negative implications. They call for modesty and measures to harness the hype.

This indicates that despite great scientific efforts (bias mitigation) and ethical endeavors (AI ethics) we must expect and live with some unknown or residual biases from AI systems. Rather than scaring us off, this should sharpen our attention and inspire our efforts to address biases in AI systems both scientifically and ethically. Even more, it requires a close collaboration between scientists and ethicists.

## Discussion

This article started by briefly reviewing the main types of biases in AI systems and identified a series of basic ethical issues from these biases. Then it examined some of the many ways to identify, measure, and mitigate these biases. While acknowledging these great efforts, there are (yet) no measures to eliminate all biases in AI systems. Residual biases pose inevitable epistemic challenges with profound ethical implications and issues. The article then briefly scrutinized whether the general principles, checklists, guidelines, frameworks, or regulations of ethics in AI systems could address the identified ethical issues. However, due to the unresolved epistemic challenges, it is (yet) unlikely that these general approaches will address the ethical issues of biases. Accordingly, we have to acknowledge and live with the ethical issues listed in Table 1 and Figure 4. A host of approaches in ethics and moral psychology offer support to do so. An important lesson from this study is that we have to take biases and their basic ethical issues into account when assessing and implementing AI systems.

It is important to notice that I do not claim or promote any kind of AI exceptionalism. Biases occur with all types of health decisions, and epistemic challenges with ethical implications result from very many technologies, including AI systems (167, 168). However, the hype of AI, its widespread, and partially uncritical implementation makes the ethics of biases in AI highly pertinent.

Moreover, I have not argued that biases will never be eradicated or that ethical principles or frameworks will not ever be able to address the ethical issues. I have only argued that, yet they do not.

Additionally, I have ignored a range of issues, such as global sustainability of developing algorithms. Furthermore, I have not addressed aspects like “the danger of a malevolent artificial general intelligence, machine consciousness, the reduction of social cohesion by AI ranking and filtering systems on social networking sites, the political abuse of AI systems, a lack of diversity in the AI community, links to robot ethics, the dealing with trolley problems, the weighting between algorithmic or human decision routines, “hidden” social and ecological costs of AI, to the problem of public–private-partnerships and industry-funded research” (18). These are issues for further work.

The implications listed in Table 1 are neither exhaustive nor exclusive. The ethical implications of bias in AI systems can interact and overlap. For example, the overreliance may stem from transformative and conceptual changes. Nonetheless, I believe that the categories are relevant for addressing the ethical issues of bias in AI systems. Future work and development may refine this typology.

Moreover, the review is not exhaustive when it comes to bias mitigation measures or ethical principles and frameworks for AI. The latter has more than 3,680,000 references in Google Scholar. Many more relevant references could have been added, e.g., on intersectionality frameworks applied to AI bias and emerging algorithmic auditing standards (88, 169). Regulatory measures could also have been included, such as the EU AI Act, which in Article 15 addresses bias and refers to the ethical principles of fairness, accountability, transparency, and privacy (170–172). However, they do not provide specific measures and practices to address the ethical issues of biases in AI.

As acknowledged in the introduction, biases may have morally good effects. It is argued that bias can be helpful or contribute to balance injustice (52, 173, 174) and that biases may be corrective: “bias itself might be used to counter the effects of certain other types of bias risks” (173) e.g., in order to reduce risk. There are also ways that AI can be used to omit or reduce human biases. For example, AI can be used for preference-identification and predictions, as humans are bad at anticipating and deliberate on future events due to various biases (175, 176). Even if some biases are good, we need to differentiate the good from the bad, i.e., we need to identify the negative implications of biases in AI (and balance them against the positive ones as well as the benefits from the AI systems as such). Reviewing the morally good aspects of bias in AI is beyond the scope of this study and warrants a separate investigation.

Moreover, the scope of this study, and its examples, has been limited to healthcare. While the findings may be relevant for other fields of AI applications or for AI bias in general, further studies are needed to investigate its generalizability and transferability. Such studies can benefit from comparative studies with and within other fields, such as criminal justice (85, 177, 178).

As acknowledged, the study of bias may itself be biased. The literature has identified stakeholders to be “bias apologists” and “bias deniers” (179). This may challenge the work on acknowledging and addressing the biases and its ethical implications.

## Conclusion

The brief review of the vast number of biases in AI systems identified three main types of bias: input bias, system bias, and application bias. These biases pose a series of basic ethical challenges: injustice, bad output/outcome, loss of autonomy, transformation of basic concepts and values, and loss of accountability (IBATA). Reviewing the many ways to identify, measure, and mitigate these biases demonstrated great efforts to reduce biases and their ethical implications. However, at present they are not able to eliminate all biases. Some biases remain unknown, and residual biases pose inevitable epistemic challenges with profound ethical implications and issues. Investigating whether the general principles, checklists, guidelines, frameworks, or regulations of AI ethics could address the identified ethical issues with bias ends negative as the ethical issues are profound, diverse, and complex. Instead, it is suggested that we have to live with the (residual) ethical issues of biases in AI systems. A host of approaches in ethics and moral psychology offer support to do so.

Few technologies are flawless. Avoiding all ethical issues of AI is impossible. However, the task is to maximize the benefits and minimize the harms—and to provide so much knowledge about both benefits and harms as possible. Therefore, we must identify and mitigate as many biases as possible and strive to reveal the consequences of those that cannot be avoided.

The epistemic and ethical challenges with biases in AI systems both should sharpen our attention and inspire our efforts both scientifically and ethically. Even more, it requires a close collaboration between scientists and ethicists.

Overall, we must strive to use this powerful tool to obtain our goals instead of letting it dictate our values.
