# Supplementary material for: Biases in AI: acknowledging and addressing the inevitable ethical issues
Source: Front Digit Health. 2025 Aug 20;7:1614105. doi: 10.3389/fdgth.2025.1614105 (PMC12405166; doi:10.3389/fdgth.2025.1614105)
Supplement: Supplementary file 1 [file Datasheet1.pdf]

Supplementary Material

1 Supplementary Table 1 Overview of Input Biases. Based on Schwartz R, Schwartz R, Vassilev A, Greene K, Perine L, Burt A, et al. Towards a Standard for Identifying and Managing Bias in Artificial Intelligence. US Department of Commerce, National Institute of Standards and Technology (NIST)(2022).

Bias	Explanation/ Definition
Automation complacency	When humans over-rely on automated systems or have their skills attenuated by such over-reliance (e.g., spelling and autocorrect or spellcheckers).
Consumer bias	Arises when an algorithm or platform provides users with a new venue within which to express their biases, and may occur from either side, or party, in a digital interaction
Mode confusion bias	When modal interfaces confuse human operators, who misunderstand which mode the system is using, taking actions which are correct for a different mode but incorrect for their current situation.
Cognitive bias	A broad term referring generally to a systematic pattern of deviation from rational judgement and decision-making.
Anchoring effect	A cognitive bias, the influence of a particular reference point or anchor on people's decisions
Availability heuristic	A mental shortcut whereby people tend to overweight what comes easily or quickly to mind (Availability bias)
Confirmation bias	A cognitive bias where people tend to prefer information that aligns with, or confirms, their existing beliefs
Dunning–Kruger effect	A cognitive bias, the tendency of people with low ability in a given area or task to overestimate their self-assessed ability
Implicit bias	An unconscious belief, attitude, feeling, association, or stereotype that can affect the way in which humans process information, make decisions, and take actions.
Loss of situational awareness	When automation leads to humans being unaware of their situation such that, when control of a system is given back to them in a situation where humans and machines cooperate, they are unprepared to assume their duties.

User interaction bias	Arises when a user imposes their own self-selected biases and behavior during interaction with data, output, results etc.

Supplementary Table 2 Overview of System Biases. Based on Schwartz R, Schwartz R, Vassilev A, Greene K, Perine L, Burt A, et al. Towards a Standard for Identifying and Managing Bias in Artificial Intelligence. US Department of Commerce, National Institute of Standards and Technology (NIST)(2022)

Level	Bias	Explanation / Definition
**SELECTION AND SAMPLING**	Data generation bias	Arises from the addition of synthetic or redundant data samples to a dataset
	Detection bias	Systematic differences between groups in how outcomes are determined and may cause an over- or underestimation of the size of the effect
	Ecological fallacy	Occurs when an inference is made about an individual based on their membership within a group
	Evaluation bias	Arises when the testing or external benchmark populations do not equally represent the various parts of the user population or from the use of performance metrics that are not appropriate for the way in which the model will be used
	Exclusion bias	When specific groups of user populations are excluded from testing and subsequent analyses
	Measurement bias	Arises when features and labels are proxies for desired quantities, potentially leaving out important factors or introducing group or input-dependent noise that leads to differential performance
	Popularity bias	A form of selection bias that occurs when items that are more popular are more exposed and less popular items are under-represented
	Population bias	Systematic distortions in demographics or other user characteristics between a population of users represented in a dataset or on a platform and some target population

	Representation bias	Arises due to non-random sampling of subgroups, causing trends estimated for one population to not be generalizable to data collected from a new population
	Simpson's Paradox	A statistical phenomenon where the marginal association betweentwo categorical variables is qualitatively different from the partial association between the same two variables after controlling for one or more other variables
	Temporal bias	Bias that arises from differences in populations and behaviors over time
	Uncertainty bias	Arises when predictive algorithms favor groups that are better represented in the training data, since there will be less uncertainty associated with those predictions
**PROCESSING/ VALI-DATATION**	Amplification bias	Arises when the distribution over prediction outputs is skewed in comparison to the prior distribution of the prediction target
	Inherited bias	Arises when applications that are built with machine learning are used to generate inputs for other machine learning algorithms.
	Error propagation bias	Arises when applications that are built with machine learning are used to generate inputs for other machine learning algorithms. If the output is biased in any way, this bias may be inherited by systems using the output as input to learn other models
	Model selection bias	The bias introduced while using the data to select a single seemingly "best" model from a large set of models employing many predictor variables
	Survivorship bias	The tendency for people to focus on the items, observations, or people that "survive" or make it past a selection process, while overlooking those that did not
**USE AND INTERPRE-TATION**	Activity bias	A type of selection bias that occurs when systems/platforms get their training data from their most active users, rather than those less active
	Concept drift bias	Use of a system outside the planned domain of application, and a common cause of performance gaps between laboratory settings and the real world

	Emergent bias	Use of a system outside the planned domain of application, and a common cause of performance gaps between laboratory settings and the real world
	Content production bias	Arises from structural, lexical, semantic, and syntactic differences in the contents generated by users
	Data dredging	A statistical bias in which testing huge numbers of hypotheses of a dataset may appear to yield statistical significance even when the results are statistically Nonsignificant
	Feedback loop bias	Effects that may occur when an algorithm learns from user behavior and feeds that behavior back into the model
	Linking bias	Arises when network attributes obtained from user connections, activities, or interactions differ and misrepresent the true behavior of the users

Supplementary Table 3 Overview of Application Biases. Based on Schwartz R, Schwartz R, Vassilev A, Greene K, Perine L, Burt A, et al. Towards a Standard for Identifying and Managing Bias in Artificial Intelligence. US Department of Commerce, National Institute of Standards and Technology (NIST)(2022)

**Level**	**Bias**	**Explanation/ Definition**
**Individual**	Behavioral bias	Systematic distortions in user behavior across platforms or contexts, or across users represented in different datasets
	Interpretation bias	A form of information processing bias that can occur when users interpret algorithmic outputs according to their internalized biases and views
	Rashomon effect or principle	Refers to differences in perspective, memory and recall, interpretation, and reporting on the same event from multiple persons or witnesses
	Selective adherence bias	Decision-makers' inclination to selectively adopt algorithmic advice when it matches their pre-existing beliefs and stereotypes

	Streetlight effect	A bias whereby people tend to search only where it is easiest to look
	Annotator reporting bias	When users rely on automation as a heuristic replacement for their own information seeking and processing
	Human reporting bias	When users rely on automation as a heuristic replacement for their own information seeking and processing
	Presentation bias	Biases arising from how information is presented on the Web, via a user interface, due to rating or ranking of output, or through users' own self-selected, biased interaction
	Ranking bias	A form of anchoring bias. The idea that top-ranked results are the most relevant and important and will result in more clicks than other results
**Group**	Groupthink bias	A psychological phenomenon that occurs when people in a group tend to make non-optimal decisions based on their desire to conform to the group, or fear of dissenting with the group.
	Funding bias	Arises when biased results are reported in order to support or satisfy the funding agency or financial supporter of the research study
	Deployment bias	Arises when systems are used as decision aids for humans, since the human intermediary may act on predictions in ways that are typically not modeled in the system
	Sunk cost fallacy	A human tendency where people opt to continue with an endeavor or behavior due to previously spent or invested resources, such as money, time, and effort, regardless of whether costs outweigh benefits
